# Empirical assessment of alternative methods for identifying seasonality in observational healthcare data

**DOI:** 10.1186/s12874-022-01652-3

**Published:** 2022-07-02

**Authors:** Anthony Molinaro, Frank DeFalco

**Affiliations:** Janssen Research and Development, 1125 Trenton Harbourton Rd, Titusville, NJ 08560 USA

**Keywords:** ACHILLES, ARIMA, CASTOR, Classification, Common data model, Cyclical, Observational data, OHDSI, OMOP CDM, R, Seasonality, Time series

## Abstract

**Background:**

Seasonality classification is a well-known and important part of time series analysis. Understanding the seasonality of a biological event can contribute to an improved understanding of its causes and help guide appropriate responses. Observational data, however, are not comprised of biological events, but timestamped diagnosis codes the combination of which (along with additional requirements) are used as proxies for biological events. As there exist different methods for determining the seasonality of a time series, it is necessary to know if these methods exhibit concordance. In this study we seek to determine the concordance of these methods by applying them to time series derived from diagnosis codes in observational data residing in databases that vary in size, type, and provenance.

**Methods:**

We compared 8 methods for determining the seasonality of a time series at three levels of significance (0.01, 0.05, and 0.1), against 10 observational health databases. We evaluated 61,467 time series at each level of significance, totaling 184,401 evaluations.

**Results:**

Across all databases and levels of significance, concordance ranged from 20.2 to 40.2%. Across all databases and levels of significance, the proportion of time series classified seasonal ranged from 4.9 to 88.3%. For each database and level of significance, we computed the difference between the maximum and minimum proportion of time series classified seasonal by all methods. The median within-database difference was 54.8, 34.7, and 39.8%, for *p* < 0.01, 0.05, and 0.1, respectively.

**Conclusion:**

Methods of binary seasonality classification when applied to time series derived from diagnosis codes in observational health data produce inconsistent results. The methods exhibit considerable discord within all databases, implying that the discord is a result of the difference between the methods themselves and not due to the choice of database. The results indicate that researchers relying on automated methods to assess the seasonality of time series derived from diagnosis codes in observational data should be aware that the methods are not interchangeable and thus the choice of method can affect the generalizability of their work. Seasonality determination is highly dependent on the method chosen.

**Supplementary Information:**

The online version contains supplementary material available at 10.1186/s12874-022-01652-3.

## Background

Events of interest (EOI) for which changes in frequency of occurrence follow a repeatable pattern based on calendar date are considered seasonal. Discovering whether an EOI is more likely to occur on a particular calendar date can contribute to an improved understanding of the EOI, its causes, and appropriate responses. Given a visualization of the frequency of occurrence of an EOI, the human eye can often determine whether a repeatable pattern, such as seasonality, exists. However, detection by eye is not feasible when working with large volumes of data containing thousands of potential EOI, therefore automated statistical methods must be employed. Necessarily, when relying on automated methods to discover true patterns, the existence of alternative methods and whether they are concordant should be known prior to investigation.

Observational data is patient level data comprised of prescription and health insurance claims, billing, and electronic health records. These data are assessed in various ways to determine whether they are appropriate for a given analysis. Healthcare researchers often attempt to assess the seasonality of an EOI by employing a variety of methods [1–5]. Given the existence of alternative methods, it is necessary to know if these methods exhibit concordance. To date, an analysis of the concordance of popular methods of seasonality classification has not been conducted. Given the existence of many different alternative methods of seasonality classification and the dearth of published literature reporting their discordance, our expectation was that the methods would be largely concordant. In this study we seek to determine the concordance of these methods by applying them to time series derived from diagnosis codes in observational data.

## Methods

### Data sources

We used a total of 10 databases varying in size, provenance, and type, to ensure our results are not database dependent. Table 1 lists each database, abbreviation, the number of time series evaluated, the number of people, database type, and period covered. More detailed descriptions of the databases can be found in the appendix.

**Table 1:** Databases used in this study

Database	Time Series	People	Type	Period
Premier Healthcare Database (PHD)	6635	264 M	Hospital Charge	2000–2021
Japan Medical Data Center (JMDC)	2956	13 M	Claims	2000–2021
Optum Electronic Health Records (EHR)	12,102	101 M	Electronic Health Records	2007–2021
IBM MarketScan® Commercial Claims and Encounters (CCAE)	11,051	157 M	Claims	2000–2021
IQVIA Disease Analyzer - France (FRA)	896	4 M	General Practitioner	2016–2021
IQVIA Disease Analyzer – Germany (GER)	3208	31 M	General Practitioner	2011–2021
IQVIA Australian Longitudinal Patient Data (AUS)	408	5 M	General Practitioner	1996–2021
IBM MarketScan® Medicare Supplemental and Coordination of Benefits (MDCR)	6596	10 M	Claims	2000–2021
IBM MarketScan® Multi-State Medicaid (MDCD)	6478	31 M	Claims	2006–2021
Optum Clinformatics Extended Data Mart – Date of Death (DOD)	11,137	91 M	Claims	2000–2021

### Data conversion and time series creation

Each database had been previously converted to the Observational Medical Outcomes Partnership (OMOP) Common Data Model (CDM) [6]. The OMOP CDM organizes data into specific tables based on the type or domain of the data. The data used in this study comes from a table containing all condition occurrences where records are comprised of diagnosis codes and the corresponding dates when the codes were recorded in the data. Diagnosis codes in this table have been standardized to a unique identifier specified in the OMOP CDM vocabulary called a concept identifier.

As this study is concerned with contrasting methods of seasonality classification, it was most natural to create monthly time series objects representing how often these concept identifiers occur in the data. This was accomplished using the R programming language. An R package called ACHILLES (Automated Characterization of Health Information at Large-scale Longitudinal Evidence Systems) [7], was used to aggregate the records associated with each condition concept identifier into monthly counts. An R package called CASTOR (Characterization and Analysis of Statistical Time series Of Real-world data) [8], was developed to transform these counts into proportions and create time series. The numerator of the proportion consists of the number of people (per thousand), with the condition concept identifier in each month, while the denominator consists of the number of people with an observation period spanning said month. For a concept to be eligible to be converted into a time series, we require at least four complete years (i.e., 12 months of counts each year) of data.

### Methods of binary seasonality classification

We evaluated 8 alternative methods for determining the seasonality of a time series at three levels of significance (0.01, 0.05, and 0.1), against 10 databases. The methods were implemented using R packages Forecast [9] and Seastests [10, 11]. For convenience, the methods evaluated are listed in Table 2. A more detailed description of the methods can be found in the appendix.

**Table 2:** Methods Summary

METHOD NAME	ABBREVIATION	BRIEF DESCRIPTION
Edwards’ Test [12–14]	ED	Hypothesis test of a harmonic model of data using a linear combination of sine and cosine (periodic for 2nπ, thus trend removal is not required). The modeled data are fit using a Poisson generalized linear model. Seasonality is determined by evaluating the peaks and troughs of the modeled curve fit to the observed time series. Implementation in R follows [14].
Friedman’s Test [15]	FR	Hypothesis test using a non-parametric approach for comparing samples within a population or from populations with identical medians. A rank-based approach is employed to test the hypothesis of no seasonality of the ranked months. Any linear trend in the data is removed prior to testing for seasonality. Implementation in R follows [11].
ARIMA Hypothesis Test [9, 16–20]	AR	Hypothesis test to determine if the seasonal component is significant when compared to an identical ARIMA model without a seasonal component. Any linear trend in the data is removed prior to testing for seasonality. Implementation in R follows [9].
QS Test [21]	QS	Hypothesis test to determine seasonality by examining the autocorrelation of seasonal lags. The observed time series is seasonal if positive autocorrelations at either lag 12 or 24 are significant. Any linear trend in the data is removed prior to testing for seasonality. Implementation in R follows [11].
ETS Hypothesis Test [9, 16–20]	ET	Hypothesis test to determine if the seasonal component is significant when compared to an identical ETS model without a seasonal component. Any linear trend in the data is removed prior to testing for seasonality. Implementation in R follows [9].
Kruskal-Wallis Test [22]	KW	Hypothesis test using a non-parametric approach to compare samples from a population. A rank-based approach is employed to test the hypothesis that the monthly data have the same mean. Any linear trend in the data is removed prior to testing for seasonality. Implementation in R follows [11].
Welch’s Test [23]	WE	Hypothesis test employing one-way ANOVA, but allowing for unequal variances amongst the groups of months. Seasonality is determined if hypothesis that the monthly means are identical is rejected. Any linear trend in the data is removed prior to testing for seasonality. Implementation in R follows [11].
Auto ARIMA Test [9, 16–20]	AA	Test based on minimizing forecast errors across different models. The observed time series is considered seasonal if the optimal ARIMA model chosen (the one that minimizes forecast error) includes a seasonal component. Any linear trend in the data is removed prior to testing for seasonality. Implementation in R follows [9].

## Experiment

The choice to perform the experiment across many databases was necessary to determine whether discordance is a property of the methods themselves or the database. As the databases vary in type, size, and provenance, method discordance between databases can and should be expected. However, if the methods are truly concordant, then at a minimum they would exhibit within-database concordance.

For each combination of database, method, significance level, and time series, we record the binary classification of seasonality. For each database and level of significance, we count the number of individual time series that are considered seasonal, compute the proportion seasonal, and compute concordance. We also record the number of times specific agreement-combinations occur. These are all within-database computations. We define concordance as unanimous agreement within a database across all methods for a given time series. Therefore, the methods are concordant when they all classify a particular time series as either seasonal or non-seasonal on a given database. For the purposes of this study, the concern is not whether an individual method considers a given time series seasonal. Rather, the desired insight is whether all methods classify a given time series the same way on a given database. The concordance calculation is necessary because even identical proportions can hide disagreement. When two methods classify a similar proportion of time series as seasonal, it is useful to know whether the proportions are comprised of the same individual time series. This is impossible to determine by mere inspection of the proportion since an identical proportion may be had by classifying the same number of completely different time series.

## Results

We evaluated 61,467 time series across 10 observational databases at three levels of significance (0.01, 0.05, and 0.1), totaling 184,401 evaluations. Visualizations and tables were generated for each combination of database, method, and significance level. In an effort to provide a concise summary of the experiment, a subset of the results which is representative of the entire experiment will be presented.

Tables 3, 4, and 5 display the proportion of time series classified seasonal by each method on all databases, for *p* < 0.05, 0.1, and 0.01, respectively. Each row represents the results for all methods against a given database at the specified level of significance. The method that classified the largest proportion of time series seasonal on a given database is highlighted in red. The method that classified the smallest proportion of time series seasonal in each database is highlighted blue. Upon examining these tables, we observe substantial within-database variation across all levels of significance. For instance, in Table 3, we see that for the PHD database, the QS method classified 30.5% of the time series seasonal, while the AA method classified 79.2% seasonal. However, the QS and AA methods are not always the methods that classify the lowest and highest proportion of time series as seasonal. The method that classifies the least or greatest proportion of time series seasonal varies by database in Table 3. The methods KW, WE, AA, ED yielded the highest proportion in at least one database, while QS, ET, AR, and ED yielded the lowest proportion in at least one database. Tables 3, 4 and 5 reveal substantial within-database variation, but the significance levels of 0.01 and 0.1 influence the methods in a way that 0.05 does not. In Table 4, the ET and ED methods classify the lowest and highest proportion of time series as seasonal for all but three databases. In Table 5, the ED and AA methods classify the lowest and highest proportion of time series as seasonal for all but one database.

**Table 3:**
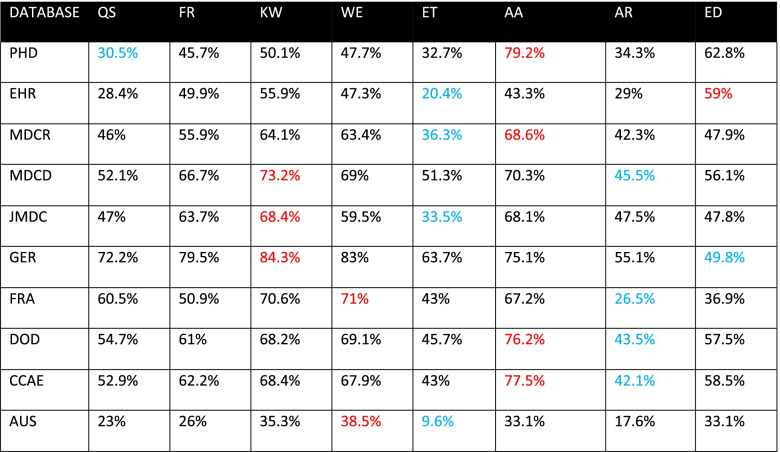
Proportion of time series classified seasonal, *p* < 0.05, blue indicates min, red indicates max

**Table 4:**
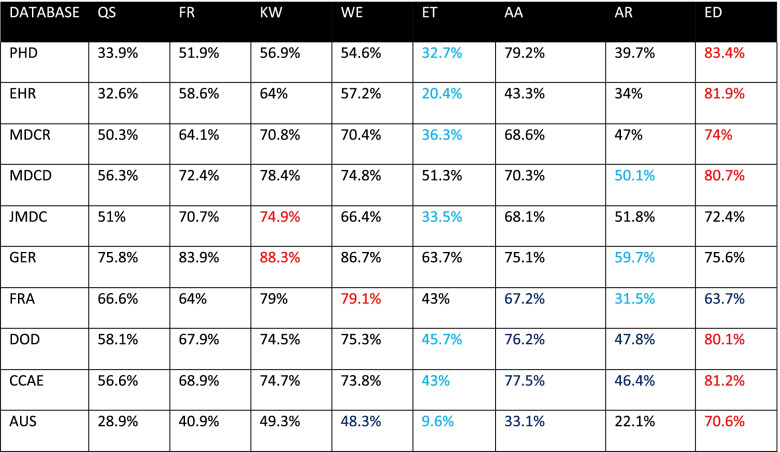
Proportion of time series classified seasonal, *p* < 0.1, blue indicates min, red indicates max

**Table 5:**
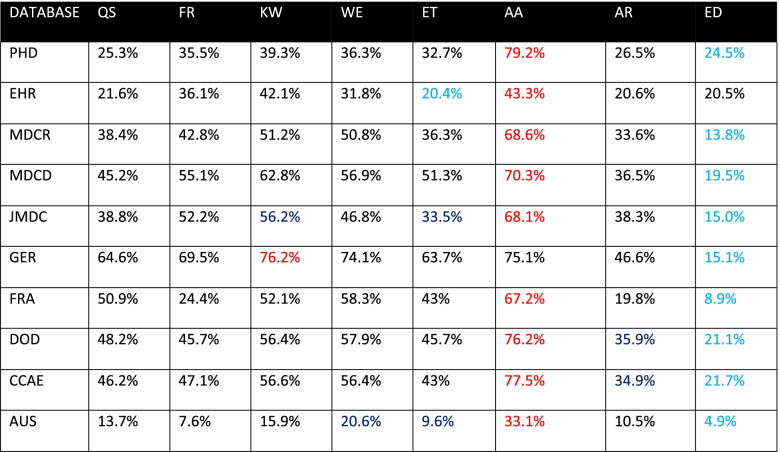
Proportion of time series classified seasonal, *p* < 0.01, blue indicates min, red indicates max

**Fig. 1:**
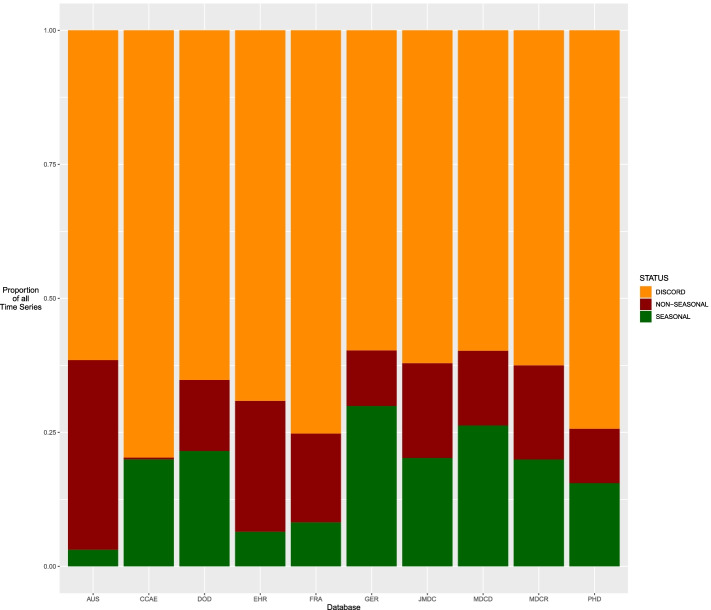
Stacked bar chart visualizing concordance by database across all significance levels

Figure 1 displays the proportion of concordance across all databases, for all methods and levels of significance. Concordance is represented by the green and red bars. The range of concordance is 20.2 to 40.2%.

For further exploration into the behavior of the individual methods, we provide the following statistics, figures, and tables from OPTUM DOD, *p* < 0.05. On this database, the methods exhibit concordance for 4307 time series; classifying 2809 as seasonal and 1498 as non-seasonal. The mean and maximum variance for the 2809 time series classified seasonal are 0.031 and 18.4, respectively. The mean and maximum variance for the 1498 time series classified non-seasonal are 0.000014 and 0.019265, respectively.

**Fig. 2:**
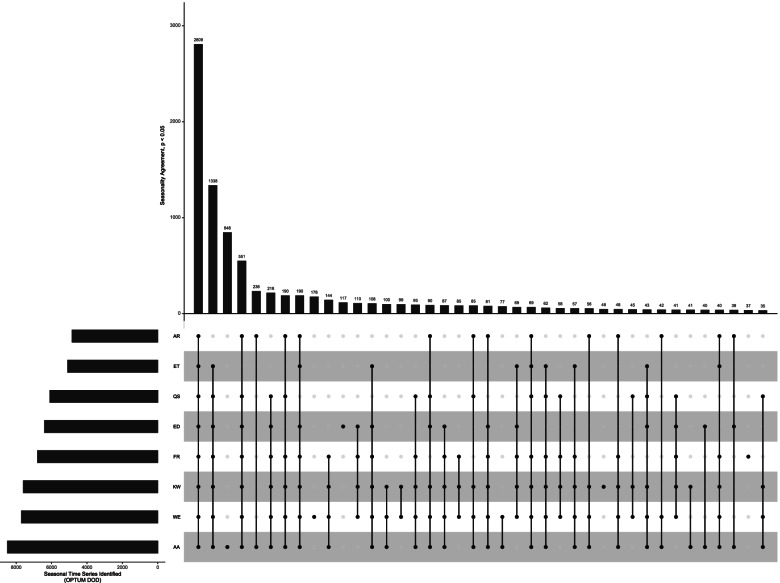
UpSetR plot visualizing 40 different method combinations of seasonality classification for OPTUM DOD, *p* < 0.05

Figure 2 is an UpsetR plot that visualizes 40 different combinations of seasonality classification on OPTUM DOD, *p* < 0.05. For any finite set S with n elements, there are 2^n^-1 non-empty subsets (combinations) of S. Since there are eight methods of seasonality classification, there are 2^8^–1 = 255 possible combinations of the eight methods. The UpsetR plot displays the top 40 combinations in terms of number of time series classified seasonal in descending order. To the left of the method names is a bar chart that shows the number of time series classified seasonal by each method. The dots with the lines through them indicate which method participated in each combination. Reading from left to right, we explain the first four combinations of methods. The first combination indicates that there were 2809 time series for which all methods agreed were seasonal. The second combination indicates that 1338 time series were classified seasonal by all methods except AR. The third combination indicates that there were 848 time series that only the AA method classified as seasonal. The fourth combination indicates that there were 551 time series classified seasonal by all methods except ET.

**Fig. 3:**
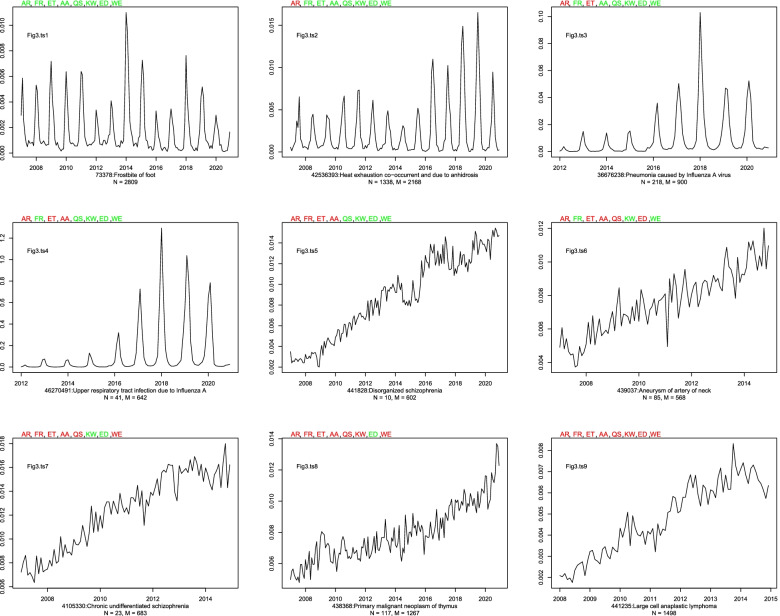
Nine time series from OPTUM DOD and their binary classification by each method, *p* < 0.05. Green method abbreviation indicates seasonal. Red method abbreviation indicates nonseasonal. *N* = The number of times the specified (green-red) combination occurred. M = The number of times any numerically similar (i.e., p seasonal and q non-seasonal) combination occurred

Figure 3 is a 3 × 3 plot of nine time series and their binary seasonality classification by each method on OPTUM DOD, *p* < 0.05. The labels of the nine time series, Fig3.ts1, …, Fig3.ts9, are located in the upper left-hand corner of each individual time series plot. Atop each time series is the abbreviation for each method. A color-coding scheme was used to indicate whether a method classified the given time series as seasonal (green) or non-seasonal (red), respectively. As per Table 2, any linear trend that appears in the original time series is removed prior to testing for seasonality. Beneath each time series is the corresponding concept identifier, name, and two different counts. The value for N represents the number of times the specified (green-red) combination occurred, while the value for M represents the number of times a numerically similar combination occurred. By a “numerically similar combination” is meant a combination with the same number of methods that agree, not necessarily the same methods. Thus, while N tells us the number of times a specific combination of k methods agree, M tells us the number of times any combination of k methods agree. For instance, AR, FR, ET, and AA all classified “Disorganized schizophrenia” (Fig3.ts5 - the center plot) as non-seasonal. Thus, four of the methods classified this time series as seasonal, while four did not. *N* = 10 implies that there were 10 time series classified as non-seasonal by this specific combination (AR, FR, ET, and AA) of four methods. M = 602 implies that there were 602 time series for which (any combination of) four methods classified as seasonal while four did not. In Fig. 3, the methods exhibit concordance for only two time series: Frostbite of foot and Large cell anaplastic lymphoma (Fig3.ts1 and Fig3.ts9, respectively).

## Discussion

The purpose of this study was to determine whether there exists concordance among different methods of binary seasonality classification when applied to time series derived from diagnosis codes in observational data. We used databases of varying size, type, and provenance to eliminate the possibility of discordance caused by mere database choice. The results of this study, as shown in Fig. 1, indicate the methods are generally inconsistent with each other, with discordance observed in 60 to 80% of time series across 10 databases. As Tables 3, 4, and 5 reveal, the methods exhibit considerable within-database variation even when only considering the proportion of time series classified as seasonal. The existence of this variation on all databases and significance levels indicates that the source of the variation is not the data, but the methods themselves.

### Sources of discord

Ultimately, the source of discord stems from the different ways in which the methods assess seasonality. While there do exist similarities, each method focuses on a different aspect of a time series to assess seasonality (Table 2). For instance, half the methods (ET, AA, AR, ED) fit a time series with a hypothetical model and test the model for seasonality, while the other half (FR, KW, WE, QS) test different aspects of a time series directly, without using a hypothesized model. To take the discussion further and generalize where we can, we make distinctions between types of concordance and types of peaks. Regarding concordance, we define “positive concordance” to be unanimous agreement among the methods that a time series is seasonal, while “negative concordance” to be unanimous agreement that a time series is non-seasonal. Therefore, for a given time series, the methods are discordant when there is neither positive concordance nor negative concordance. Regarding peaks, we say that peaks are “persistent” if they occur year after year, and they are “consistent” if they occur in the same month. We make this distinction because peaks relate to important aspects of time series analysis relevant to seasonality; specifically, variation and autocorrelation. Peaks can, of course, come in different sizes. Time series with large peaks suggest greater variation than those with small peaks. Persistent peaks (be they small or large) suggest the possibility of underlying cyclical behavior in the time series. Consistent peaks, to the extent that they are consistent, indicate autocorrelation in the time series. We’ll use Figs. 2 and 3 to navigate the remainder of the discussion.

From Fig3.ts1 (*N* = 2809) and Fig3.ts9 (*N* = 1498), we learn that the methods exhibit concordance only 4307/11,137 = 38.7% of the time. Figure 2 provides valuable insight into the extent of discord among the methods. Of the 40 unique combinations, we observe that some combinations occur more frequently than others and this is due to similarities in the testing procedure (Table 2). For instance, methods that group time series data by month and test for differences among the groups are assessing seasonality differently than methods that fit a hypothetical model and then determine seasonality by minimizing forecast error. Acknowledging the differences in how the methods assess seasonality is important not only for understanding the amount of observed discord, but in recognizing that these differences indicate a disagreement with regards to how seasonality is defined. Indeed, if the methods were highly concordant despite their contrasting approaches, we would have to concede that the contrasting approaches are ultimately just different ways of expressing the same aspect of a time series. This can be observed more clearly by exploring Fig. 3. In Fig3.ts1, …, Fig3.ts4 we observe time series that to the human eye seem seasonal and very similar. Identifying such time series as seasonal is a very old idea in time series analysis, with Beveridge [24] and Yule [25] employing harmonic functions to model time series with cyclical behavior. However, despite an obvious cyclical pattern and visual similarities, Fig3.ts2, Fig3.ts3, and Fig3.ts4, all exhibit discord. The reason being, except for the ED method, the methods are not testing for seasonality by fitting the data with harmonic functions. Thus, the different methods of seasonality assessment ultimately result in different definitions of seasonality.

As we’ve mentioned previously, the behavior of peaks plays an important role in concordance. We’ll use Fig. 3 further to explore the relationship between peaks, variation, and discord, and provide general principles as to when a method would be more likely to classify a time series as seasonal rather than non-seasonal.

### Positive concordance

Since each method assesses seasonality differently, positive concordance is only achieved when multiple conditions are simultaneously present. Persistent and consistent peaks are most important for ED, AA, AR, and ET. Peaks will result in a seasonal classification by ED, so long as there exists a sufficient difference between the peaks and troughs in the data. However, even with persistent and consistent peaks, variation (particularly among the peaks) over time can lead to a non-seasonal classification by AA, AR, or ET (Fig3.ts2, Fig3.ts3, and Fig3.ts4). Indeed, we have confirmed experimentally that we can achieve positive concordance for the time series in Fig3.ts2, Fig3.ts3, and Fig3.ts4, by removing the data prior to 2016. Since time series with persistent and consistent peaks will have high correlation between seasonal lags, they will be classified seasonal by QS. For FR, KW, and WE, most important is variation. In the absence of the prominent peaks we see in Fig3.ts1, …, Fig3.ts4, sufficient variation in the time series data can lead FR, KW, and WE to a seasonal classification (Fig3.ts6). Therefore, with regards to positive concordance we see tension among the methods in that variation may cause some methods to classify seemingly seasonal time series as non-seasonal (Fig3.ts2, Fig3.ts3, and Fig3.ts4) and seemingly non-seasonal time series as seasonal (Fig3.ts5, …, Fig3.ts8).

### Negative concordance

The relationship between negative concordance and variation is more straightforward. The time series in Fig3.ts5, …, Fig3.ts9 are similar in that one cannot determine the results of the methods by visual inspection alone (recall that any linear trend in each of the original series have been removed prior to method application). Given the similarity of the time series in Fig3.ts5, …, Fig3.ts9, it’s reasonable to wonder why they all do not exhibit negative concordance. Ultimately, time series that are constant or stationary around a constant mean with minimal variation will result in negative concordance among the methods. However, a time series with both large peaks and variation will exhibit negative concordance if there is no monthly or yearly autocorrelation (for instance, a time series generated from N(μ,σ^2^)). As was noted in the Results section, the 1498 time series for which the methods exhibit negative concordance report a mean variance of 0 to four decimal places.

### Generalization and limitations

We’ve explained general scenarios in which we can expect negative and positive concordance, but further generalization is more difficult. As Fig. 3 reveals, there are thousands of different combinations of discord (M = 2168, …, 1267) for each time series, making it difficult to predict which particular combination of discord to expect based on visual inspection of the time series alone. However, an immediate consequence of this study is that researchers using different methods are implicitly defining seasonality differently. Given the discordance between the methods, researchers relying on different methods are likely to encounter different results, thus leading to conflicting understanding of the seasonality of a time series.

Finally, we note that the study and evaluation of methods was limited to 10 observational databases and eight methods of binary seasonality classification. Different results may have been observed by modifying one or more of the design choices. As was explained in the Discussion section, aspects of a time series that influence seasonality classification include variance, autocorrelation, peak persistence, and peak consistence. Time series constructed to influence one or more of those aspects could influence concordance. We chose 10 observational databases. Perhaps adding dozens or hundreds of other databases would reveal different levels of concordance among the methods. Similarly, we chose 8 methods of binary seasonality classification. A different group of methods may have resulted in different levels of concordance.

## Conclusion

The results of this study indicate that the determination of the seasonality of a time series is highly dependent on the method chosen. The methods are not interchangeable and lead to vastly different results within the same database and across significance levels. Researchers investigating seasonality with these methods must be aware that their results are not generalizable to other methods. Researchers investigating seasonality with these methods should also be aware that their choice of method implies how they implicitly define seasonality in their study. Consequently, the method of seasonality classification chosen should be listed as a limitation of a study. The results of this study indicate that while seasonality may be intuitively understood, it is not well defined with regards to automated statistical tests.

## Supplementary Information

**Additional file 1: Appendix 1.** Detailed database descriptions.

**Additional file 2: Appendix 2.** Description of data: Detailed statistical method descriptions [26, 27].

**Additional file 3: upsetRplots.zip.** All 30 UpsetR plots.

## Data Availability

– https://products.premierinc.com/applied-sciences – https://www.ibm.com/products/marketscan-research-databases – https://www.iqvia.com/ – https://www.jmdc.co.jp/en/jmdc-claims-database/ – https://www.optum.com/business/about/data-analytics-technology.html – https://github.com/OHDSI/Achilles – https://github.com/OHDSI/Castor – https://github.com/OHDSI/CommonDataModel – https://ohdsi.github.io/TheBookOfOhdsi/ – https://cran.r-project.org/web/packages/forecast/index.html – https://cran.r-project.org/web/packages/seastests/index.html – https://cran.r-project.org/web/packages/UpSetR/index.html The databases used in this study are all commercial databases licensed from IBM, Optum, Iqvia, and JMDC, respectively.

## Abbreviations

Methods: AA: Auto ARIMA Test
AR: ARIMA Hypothesis Test
ED: Edwards’ Test
EOI: Events of interest
ET: ETS Hypothesis Test
FR: Friedman’s Test
KW: Kruskal-Wallis Test
QS: QS Test
WE: Welch’s Test
Databases: MDCR: IBM MarketScan® Medicare Supplemental and Coordination of Benefits
MDCD: IBM MarketScan® Multi-State Medicaid
CCAE: IBM MarketScan® Commercial Claims and Encounters
FRA: IQVIA Disease Analyzer – France
GER: IQVIA Disease Analyzer – Germany
AUS: IQVIA Australian Longitudinal Patient Data
JMDC: Japan Medical Data Center
EHR: Optum Electronic Health Records
DOD: Optum Clinformatics Extended Data Mart - Date of Death
PHD: Premier Healthcare Database
