# Supplementary material for: Empirical assessment of alternative methods for identifying seasonality in observational healthcare data
Source: BMC Med Res Methodol. 2022 Jul 2;22:182. doi: 10.1186/s12874-022-01652-3 (PMC9250712; doi:10.1186/s12874-022-01652-3)
Supplement: Supplementary file 1 — Additional file 1: Appendix 1. Detailed database descriptions. [file 12874_2022_1652_MOESM1_ESM.docx]

**APPENDIX 1: DETAILED DATABASE DESCRIPTIONS**

## Premier Healthcare

Premier Healthcare Database (PHD) is a nationally representative all-payer US hospital database that houses data on the inpatient and outpatient visits from non-profit, non-governmental and community and teaching hospitals and health systems. The data represent 1 in 5 inpatient hospital stays in the US. It is a visit-centric, billing database where each visit is linked with a unique billing record. The database contains information on medications, laboratory procedures, diagnostic procedures, and diagnoses with day of service for medications and procedures.

## Japan Medical Data Center

JMDC database consists of data from 60 Society-Managed Health Insurances covering workers aged 18 to 65 and their dependents (children younger than 18 years old and elderly people older than 65 years old). Those aged 66 or older are less representative as compared with whole population in the nation. When estimated among the people who are younger than 66 years old, the proportion of children younger than 18 years old in JMDC is approximately the same as the proportion in the whole nation. JMDC data includes data on membership status of the insured people and claims data provided by insurers under contract. Claims data are derived from monthly claims issued by clinics, hospitals and community pharmacies. The number of claims issued and added to JMDC database is about 800,000 per month. The size of JMDC population is 1.9 million, 1.5% of about 120 million people in the whole nation..

## Optum Electronic Health Records

Optum's longitudinal EHR repository is derived from dozens of healthcare provider organizations in the United States, that include more than 700 Hospitals and 7000 Clinics; treating more than 102 million patients receiving care in the United States. The data is certified as de-identified by an independent statistical expert following HIPAA statistical de-identification rules, and managed according to Optum(R) customer data use agreements. Clinical, claims and other medical administrative data is obtained from both Inpatient and Ambulatory electronic health records (EHRs), practice management systems and numerous other internal systems. Information is processed, normalized, and standardized across the continuum of care from both acute inpatient stays and outpatient visits. Optum(R) data elements include: demographics, medications prescribed and administered, immunizations, allergies, lab results (including microbiology), vital signs and other observable measurements, clinical and inpatient stay administrative data and coded diagnoses and procedures. In addition, Optum(R) uses natural language processing (NLP) computing technology to transform critical facts from physician notes into usable datasets. The NLP data provides detailed information regarding signs and symptoms, family history, disease related scores (i.e. RAPID3 for RA, or CHADS2 for stroke risk), genetic testing, medication changes, and physician rationale behind prescribing decisions that might never be recorded in the EHR.

## IBM MarketScan® Commercial Claims and Encounters

IBM MarketScan Commercial Database (CCAE) represent data from individuals enrolled in United States employer-sponsored insurance health plans. The data includes adjudicated health insurance claims (e.g. inpatient, outpatient, and outpatient pharmacy) as well as enrollment data from large employers and health plans who provide private healthcare coverage to employees, their spouses, and dependents. Additionally, it captures laboratory tests for a subset of the covered lives. This administrative claims database includes a variety of fee-for-service, preferred provider organizations, and capitated health plans. The major data elements contained within this database are outpatient pharmacy dispensing claims (coded with National Drug Codes (NDC), inpatient and outpatient medical claims which provide procedure codes (coded in CPT-4, HCPCs, ICD-9-CM or ICD-10-PCS) and diagnosis codes (coded in ICD-9-CM or ICD-10-CM). The data also contain selected laboratory test results (those sent to a contracted thirds-party laboratory service provider) for a non-random sample of the population (coded with LOINC codes).

## IQVIA Disease Analyzer – France

The IQVIA Disease Analyzer (DA) France database consists of data collected from French general practitioner (GP) offices for all ages. Mostly primary care physician data however some data from specialty practices (where practices are electronically connected to each other) and some lab data is included. Key attributes include demographics, prescriptions as prescribed at brand level, diagnosis, lab measurements, actions (e.g. referrals, sick notes).

## IQVIA Disease Analyzer – Germany

The IQVIA Disease Analyzer (DA) Germany database consists of data collected from physician practices and medical centers for all ages. Mostly primary care physician data however some data from specialty practices (where practices are electronically connected to each other) and some lab data is included. Key attributes include demographics, prescriptions as prescribed at brand level, diagnosis, lab measurements, actions (e.g. referrals, sick notes).

## IQVIA Australian Longitudinal Patient Data

Australia EMR source data contains medical history, allergies, immunizations, contacts and related to them medical events (test results, diagnoses, biometrics, prescriptions).

## IBM MarketScan® Medicare Supplemental and Coordination of Benefits

IBM MarketScan Medicare Supplemental Database (MDCR) represents health services of retirees in the United States with employer-sponsored Medicare supplemental coverage through privately insured fee-for-service, point-of-service, or capitated health plans. These data include adjudicated health insurance claims (e.g. inpatient, outpatient, and outpatient pharmacy). Additionally, it captures laboratory tests for a subset of the covered lives.

## IBM MarketScan® Multi-State Medicaid

The IBM(R) MarketScan(R) Multi-State Medicaid Database (MDCD) reflects the healthcare service use of individuals covered by Medicaid programs in numerous geographically dispersed states. The database contains the pooled healthcare experience of Medicaid enrollees, covered under fee-for-service and managed care plans. It includes records of inpatient services, inpatient admissions, outpatient services, and prescription drug claims, as well as information on long-term care. Data on eligibility and service and provider type are also included. In addition to standard demographic variables such as age and gender, the database includes variables such as federal aid category (income based, disability, Temporary Assistance for Needy Families) and race.

## Optum Clinformatics Extended Data Mart - Date of Death

Optum's Clinformatics(R) Data Mart is derived from a database of administrative health claims for members of large commercial and Medicare Advantage health plans.The database includes approximately 17-19 million annual covered lives, for a total of over 65 million unique lives over a 12 year period (1/2007 through 12/2019). Clinformatics(R) Data Mart is statistically de-identified under the Expert Determination method consistent with HIPAA and managed according to Optum(R) customer data use agreements. CDM administrative claims submitted for payment by providers and pharmacies are verified, adjudicated and de-identified prior to inclusion. This data, including patient-level enrollment information, is derived from claims submitted for all medical and pharmacy health care services with information related to healthcare costs and resource utilization. The population is geographically diverse, spanning all 50 states. Optum Clinformatics(R) Data Mart Data of Death (Optum DOD) also provides date of death (month and year only) for members with both medical and pharmacy coverage from the Social Security Death Master File (however after 2011 reporting frequency changed due to changes in reporting requirements) and location information for patients is at the US state level.
