# Supplementary material for: Empirical assessment of alternative methods for identifying seasonality in observational healthcare data
Source: BMC Med Res Methodol. 2022 Jul 2;22:182. doi: 10.1186/s12874-022-01652-3 (PMC9250712; doi:10.1186/s12874-022-01652-3)
Supplement: Supplementary file 2 — Additional file 2: Appendix 2. Description of data: Detailed statistical method descriptions [26, 27]. [file 12874_2022_1652_MOESM2_ESM.docx]

**APPENDIX 2: DETAILED METHOD DESCRIPTIONS**

# METHODS DESCRIPTION

## Edwards’ test

The Edwards' test was originally based on the approach outlined in [9] whereby a sine curve is fit and from the fitted curve, estimates of the "seasonal intensity" (peak-to-low ratio of the fitted curve) can be obtained. This approach was modified by Brookhart and Rothman in [10] due to the bias found in the Edwards estimator when dealing with rare events or events exhibiting very large or very small amounts of seasonality. The approach was further modified by Weinstein et al. [11], by using a Poisson generalized linear model to model event counts as a sinusoidal pattern along with a linear component for long-term trends. The model for the expected count of events E[Ni] in time period i is as follows:

$$\log(E[N_{i}]) = \beta_{0}+\beta_{1}\sin\theta_{i}+\beta_{2}\cos\theta_{i}+\beta_{3}\tau+\log\varphi_{i}$$

where τ is the number of months since the study start date,

$$\theta_{i}=\frac{2\pi(month\_of\_i-1)}{12}$$

and

$$\varphi_{i}=\frac{population size in period i \times number of days in period i}{365.25}$$

The ratio of peak-to-low occurrence rates of the process is given by

$$R=\frac{exp(\beta_{0}+\sqrt{\beta_{1}^{2}+\beta_{2}^{2}})}{exp(\beta_{0} - \sqrt{\beta_{1}^{2}+\beta_{2}^{2}})}$$

A value of R > 1 indicates that peaks are higher than troughs in the fitted model, meaning there is seasonality. The null hypothesis, R = 1, is tested against the alternative hypothesis that R > 1. The implementation and notation follow [11].

## Friedman’s test

The Friedman test is a non-parametric method for determining whether samples are drawn from the same population or from populations with equal medians. Friedman’s test was originally introduced in [12], where the procedure is described as the “method of ranks” because it relies on ranks of the observations rather than distributional assumptions. Let $r_{ij}$ be the rank of the i^th^ observation in the j^th^ year and $\mu_{ij} = E[r_{ij}]$. The test statistic is expressed as

$FR = \frac{k-1}{k}\sum_{i=1}^{k} \frac{n{(\bar{r}_{i}-(k+1)/2)}^{2}}{(k^{2}-1)/12}$

Where n is the number of observations in each period i, and $\bar{r}_{i}= \frac{1}{n}\sum_{j=1}^{k} r_{ij}$. The null hypothesis that there is no seasonality $H_{0}: \mu_{1}=... = \mu_{k}$ follows a $\chi^{2}$distribution with k-1 degrees of freedom. The implementation and notation follow [21,22].

## Auto ARIMA test

The auto ARIMA test uses a variation of the Hyndman-Khandakar algorithm [23], which combines unit root tests, minimization of the AICc and MLE to obtain an ARIMA model. The test considers a time series seasonal if the model chosen contains a seasonal component. The implementation follows [13,14,19,24].

## ARIMA hypothesis test

The ARIMA hypothesis test compares an optimally chosen ARIMA model with a seasonal component (chosen by using the auto ARIMA test) with an optimally chosen ARIMA model that does not contain a seasonal component (again, chosen by using the auto ARIMA test). Let N1 and N2 be the number of parameters estimated by models $ϴ_{1}$ and $ϴ_{2}$ respectively. The series is considered seasonal if the seasonal component is statistically significant by Wilks’ Theorem [25] and the test statistic is given by

$$W = 2\times[log L(ϴ_{1}) - log L(ϴ_{2})]$$

where the difference will be approximately $\chi^{2}$ with Δ = N1 - N2 degrees of freedom. The implementation follows [13,14,19,24] .

## QS test

The QS test is based on [15]. The test statistic QS is computed by examining the autocorrelation function at appropriate seasonal lags. Let n be the number of elements in the series and m the seasonal frequency (m=12 for monthly data). The test statistic is given by

$QS = n(n+2)[\frac{\hat{\rho}^{2}(m)}{n-m} + \frac{{max}^{2}(0,\hat{\rho}^{2}(2m))}{n-2m}]$

where the autocovariance is defined as $\gamma_{k}=E[y_{t+k}y_{t}]-E^{2}[y_{t}]$ and the autocorrelation is defined as $\rho_{k}= \frac{\gamma_{k}}{\gamma_{0}}$ , for the time series $y_{t}$. The probability distribution is approximately $\chi^{2}$with 2 degrees of freedom. The implementation and notation follow [21,22].

## ETS hypothesis test

The ETS test is an exponential smoother that implements a state space approach [14]. Each model consists of a measurement equation that describes the observed data, and some state equations that describe how the unobserved components or states (level, trend, seasonal) change over time [14] . Hence, these are referred to as state space models. Each state space model is labeled as ETS (Error, Trend, Seasonal) [14] . This label can also be thought of as ExponenTial Smoothing [14] . Model selection is implemented by utilizing likelihood maximization and information criterion (AIC, AICc, BIC) depending on the nature of the errors (additive or multiplicative) [14] . If a model with a seasonal component is chosen, it is tested for statistical significance using Wilks’ theorem as described in the Arima Hypothesis test above. The implementation follows [13,14,18,19,24] .

## Kruskal-Wallis test

The Kruskal-Wallis test [16] is similar to the Friedman test (same null hypothesis), but the test statistic is computed as

$$KW = \frac{N-1}{N}\sum_{i=1}^{k} \frac{n_{i}{(\bar{r}_{i}-(N+1)/2)}^{2}}{(N^{2}-1)/12}$$

where ranks are produced over the entire sample N rather than each period year. The test follows a $\chi^{2}$distribution with k-1 degrees of freedom under the null hypothesis. The implementation and notation follow [21,22].

## Welch’s test

As explained in [21], Welch’s test [17] is essentially a one-way ANOVA without repeated measures for heteroskedastic data. Let $\mu_{i}= E(y_{ij}) and \bar{y}_{i}= \frac{1}{n_{i}}\sum_{j=1}^{n_{i}} y_{ij}$. The test statistic is

$$WE = \frac{{(k-1)}^{-1}\sum_{i=1}^{k} w_{i}{( \bar{y}_{i} - w^{-1}\sum_{i=1}^{k} w_{i} \bar{y}_{i})}^{2}}{1+2(k+2){(k^{2}-1)}^{-1}\sum_{i=1}^{k} {(n_{i}-1)}^{-1}{(1-w^{-1}w_{i})}^{2}}$$

where $w_{i} = n_{i}/{(n_{i}-1)}^{-1}\sum_{j=1}^{n_{i}} {{(y}_{ij}-\bar{y}_{i})}^{2} and w = \sum_{i=1}^{k} w_{i}$. WE follows an F distribution under the null hypothesis, $H_{0}: \mu_{1}=... = \mu_{k}$ with k-1 and ${[3{(k^{2}-1)}^{-1}\sum_{i=1}^{k} {{(n}_{i}-1)}^{-1}{(1-\frac{w_{i}}{w})}^{2}]}^{-1}$ degrees of freedom. The implementation and notation follow [21,22].
