# Supplementary figures and images for: Empirical assessment of alternative methods for identifying seasonality in observational healthcare data
Source: BMC Med Res Methodol. 2022 Jul 2;22:182. doi: 10.1186/s12874-022-01652-3 (PMC9250712; doi:10.1186/s12874-022-01652-3)

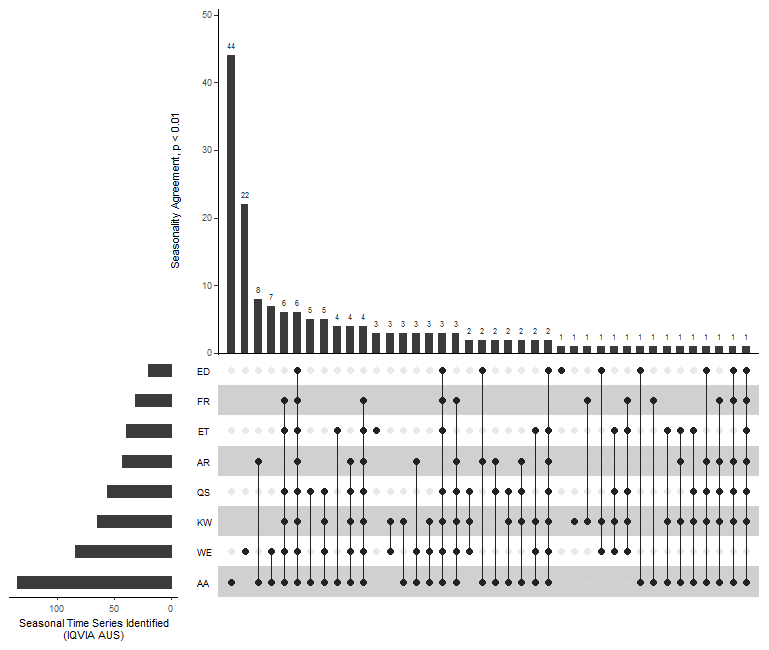

Supplement: Supplementary file 3 — Additional file 3: upsetRplots.zip. All 30 UpsetR plots. [file 12874_2022_1652_MOESM3_ESM.zip › upsetRplots/AUS01.png]

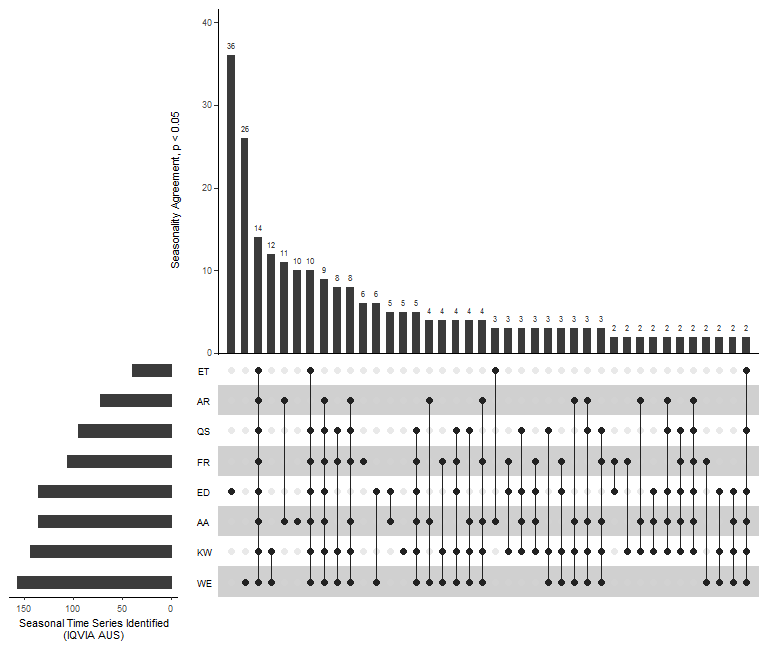

Supplement: Supplementary file 3 — Additional file 3: upsetRplots.zip. All 30 UpsetR plots. [file 12874_2022_1652_MOESM3_ESM.zip › upsetRplots/AUS05.png]

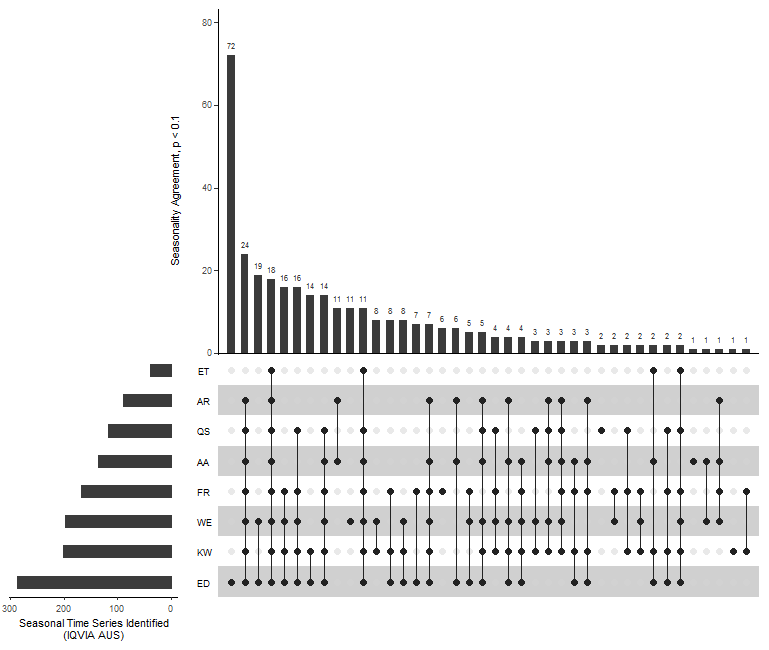

Supplement: Supplementary file 3 — Additional file 3: upsetRplots.zip. All 30 UpsetR plots. [file 12874_2022_1652_MOESM3_ESM.zip › upsetRplots/AUS1.png]

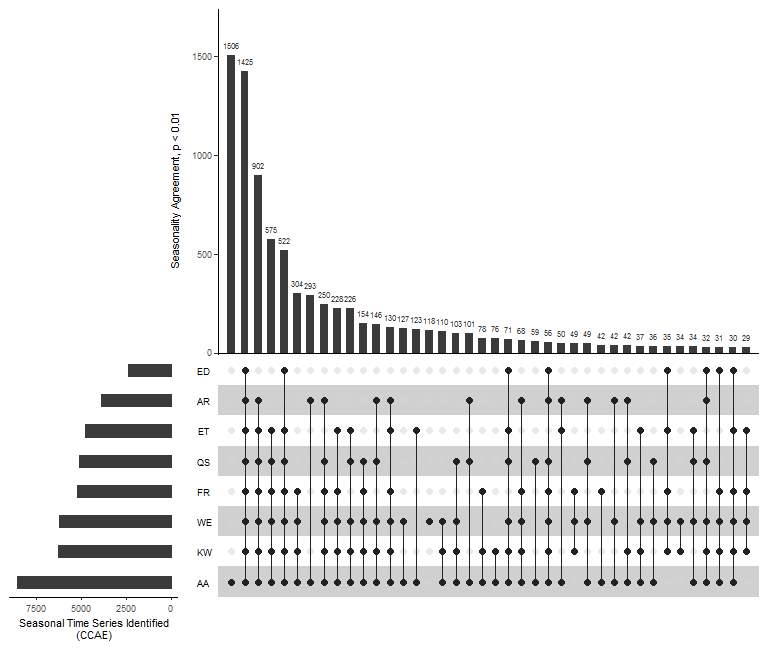

Supplement: Supplementary file 3 — Additional file 3: upsetRplots.zip. All 30 UpsetR plots. [file 12874_2022_1652_MOESM3_ESM.zip › upsetRplots/CCAE01.png]

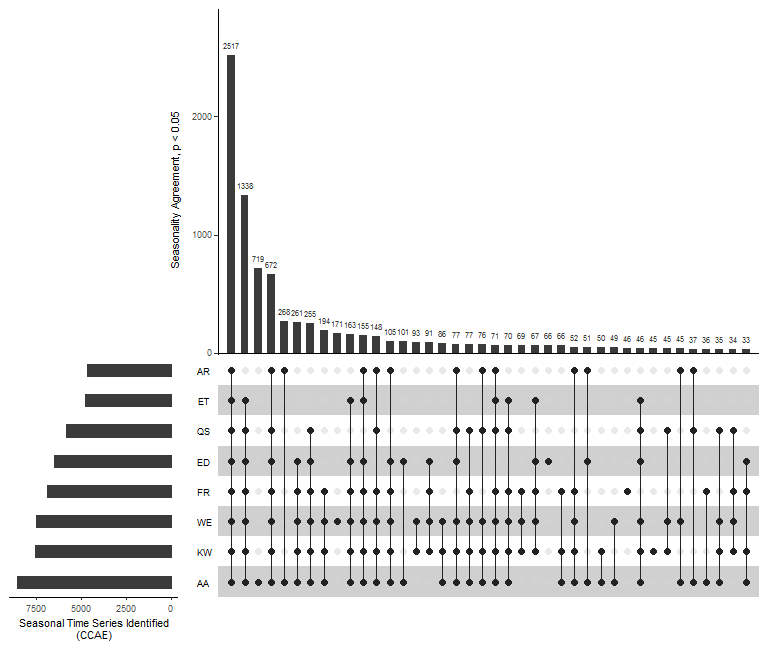

Supplement: Supplementary file 3 — Additional file 3: upsetRplots.zip. All 30 UpsetR plots. [file 12874_2022_1652_MOESM3_ESM.zip › upsetRplots/CCAE05.png]

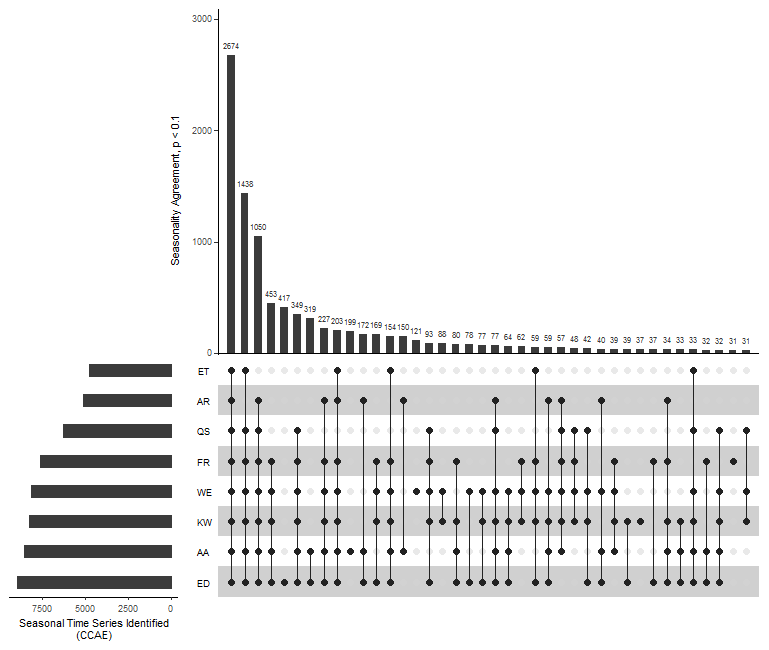

Supplement: Supplementary file 3 — Additional file 3: upsetRplots.zip. All 30 UpsetR plots. [file 12874_2022_1652_MOESM3_ESM.zip › upsetRplots/CCAE1.png]

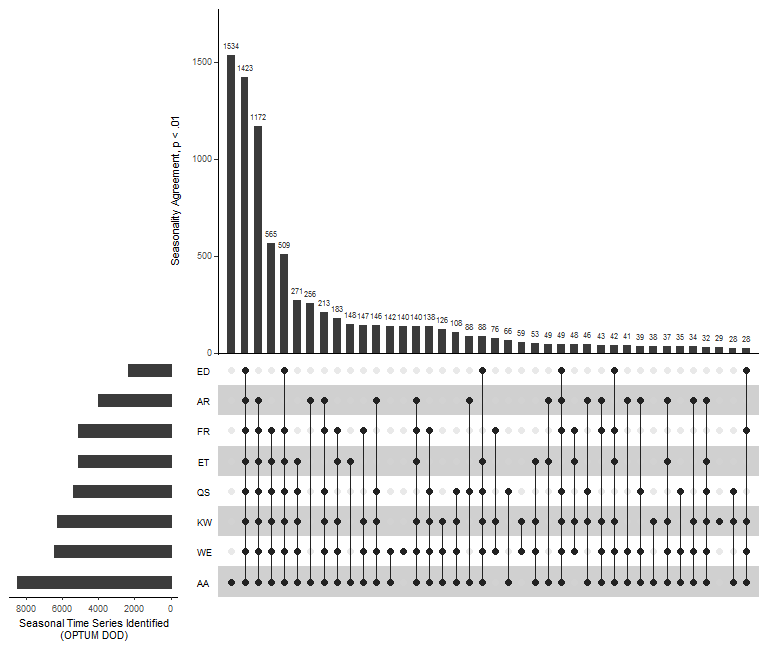

Supplement: Supplementary file 3 — Additional file 3: upsetRplots.zip. All 30 UpsetR plots. [file 12874_2022_1652_MOESM3_ESM.zip › upsetRplots/DOD01.png]

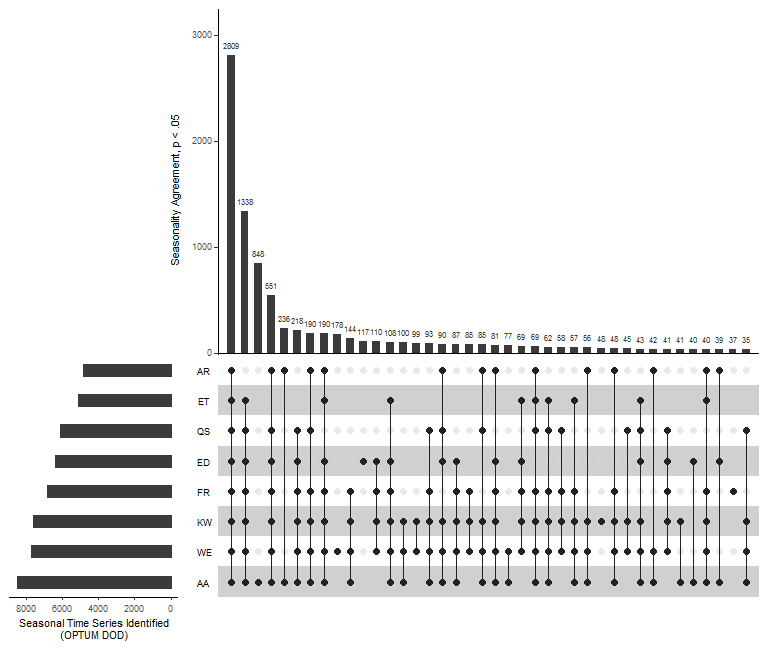

Supplement: Supplementary file 3 — Additional file 3: upsetRplots.zip. All 30 UpsetR plots. [file 12874_2022_1652_MOESM3_ESM.zip › upsetRplots/DOD05.png]

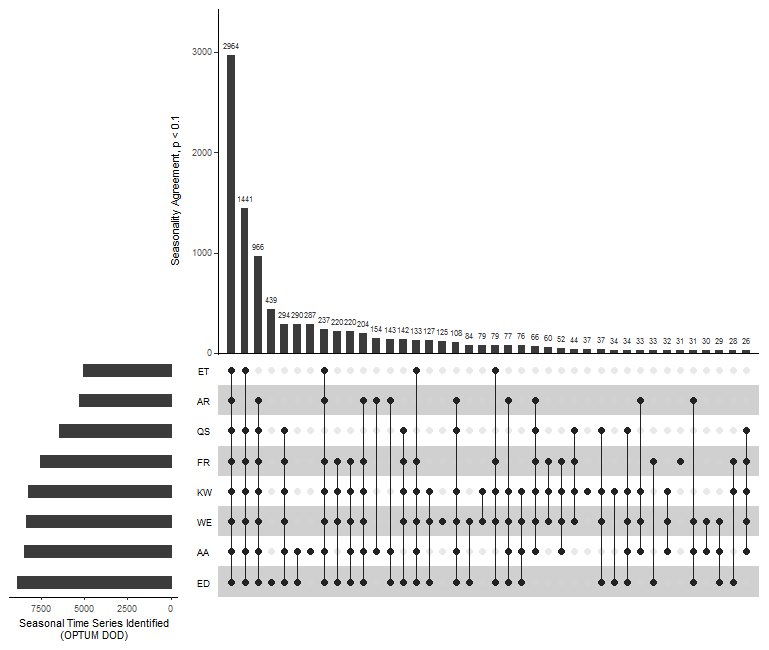

Supplement: Supplementary file 3 — Additional file 3: upsetRplots.zip. All 30 UpsetR plots. [file 12874_2022_1652_MOESM3_ESM.zip › upsetRplots/DOD1.png]

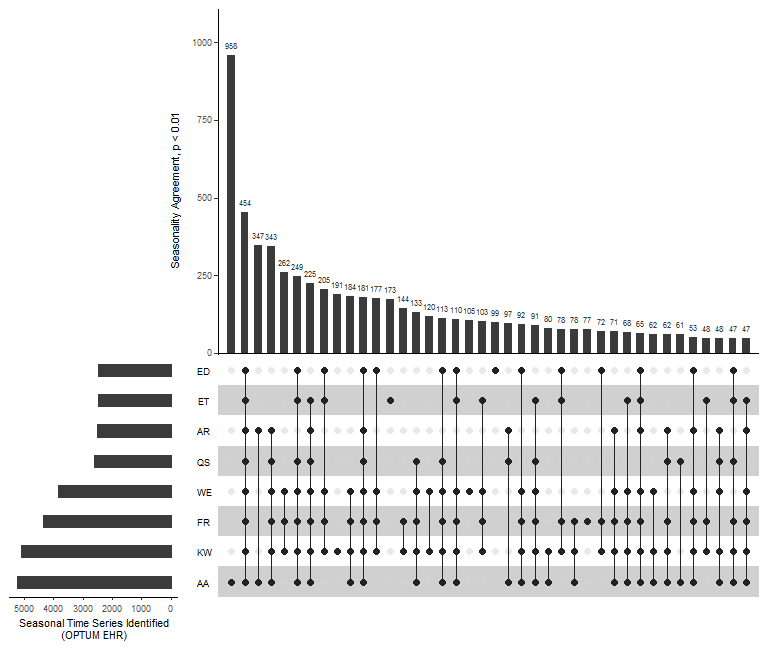

Supplement: Supplementary file 3 — Additional file 3: upsetRplots.zip. All 30 UpsetR plots. [file 12874_2022_1652_MOESM3_ESM.zip › upsetRplots/EHR01.png]

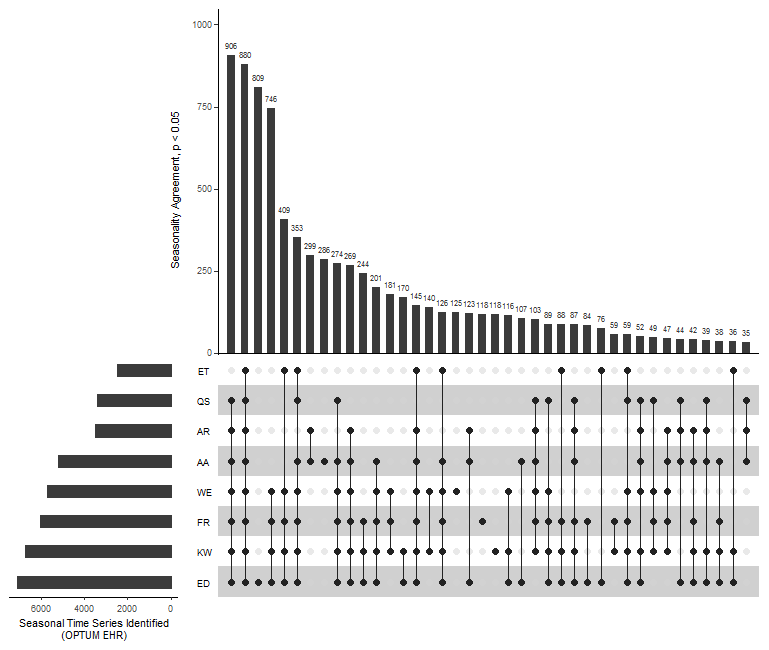

Supplement: Supplementary file 3 — Additional file 3: upsetRplots.zip. All 30 UpsetR plots. [file 12874_2022_1652_MOESM3_ESM.zip › upsetRplots/EHR05.png]

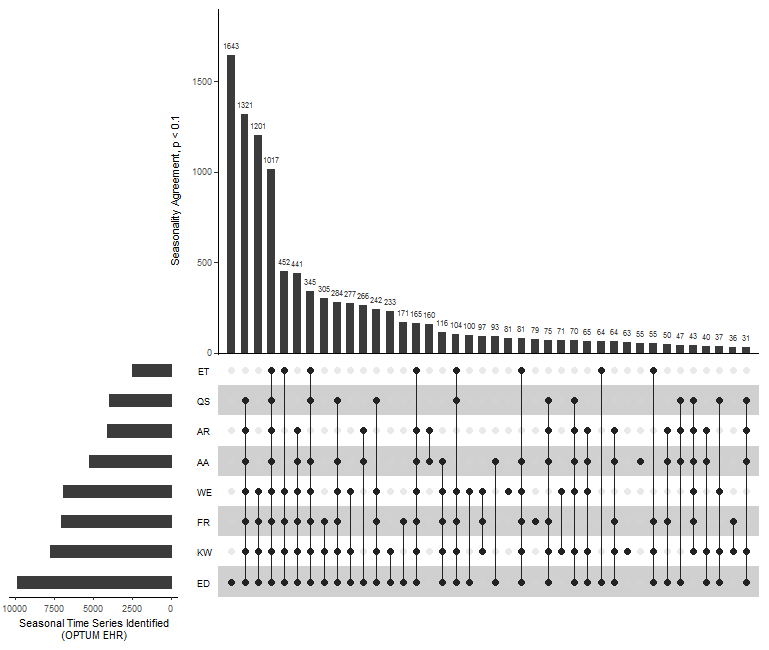

Supplement: Supplementary file 3 — Additional file 3: upsetRplots.zip. All 30 UpsetR plots. [file 12874_2022_1652_MOESM3_ESM.zip › upsetRplots/EHR1.png]

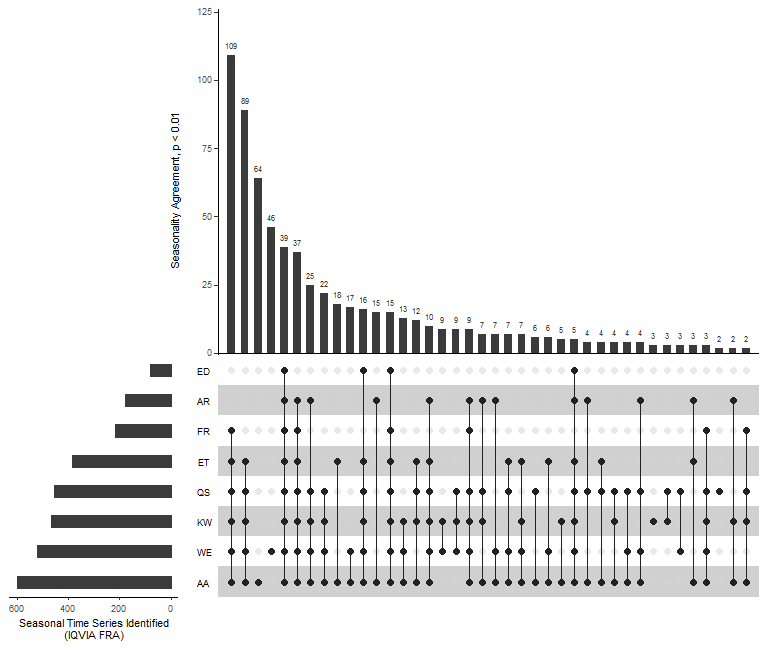

Supplement: Supplementary file 3 — Additional file 3: upsetRplots.zip. All 30 UpsetR plots. [file 12874_2022_1652_MOESM3_ESM.zip › upsetRplots/FRA01.png]

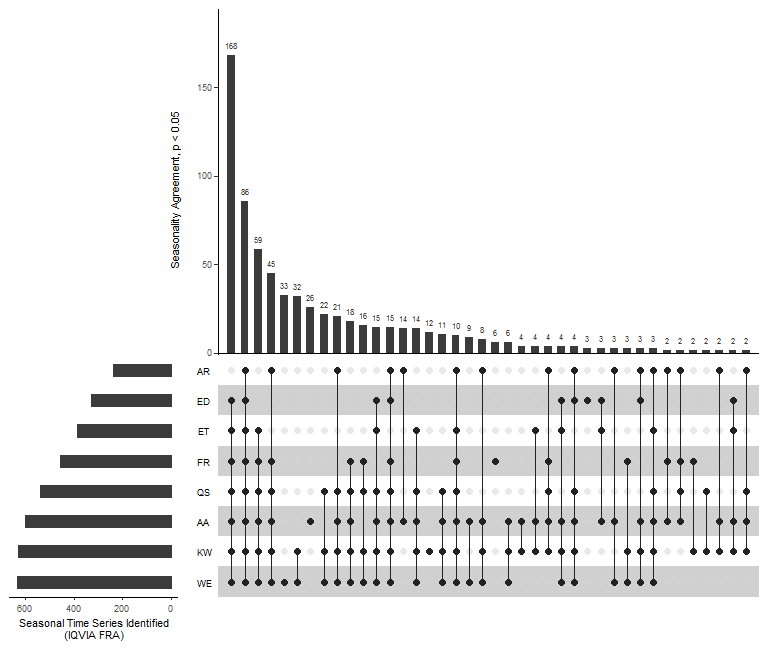

Supplement: Supplementary file 3 — Additional file 3: upsetRplots.zip. All 30 UpsetR plots. [file 12874_2022_1652_MOESM3_ESM.zip › upsetRplots/FRA05.png]

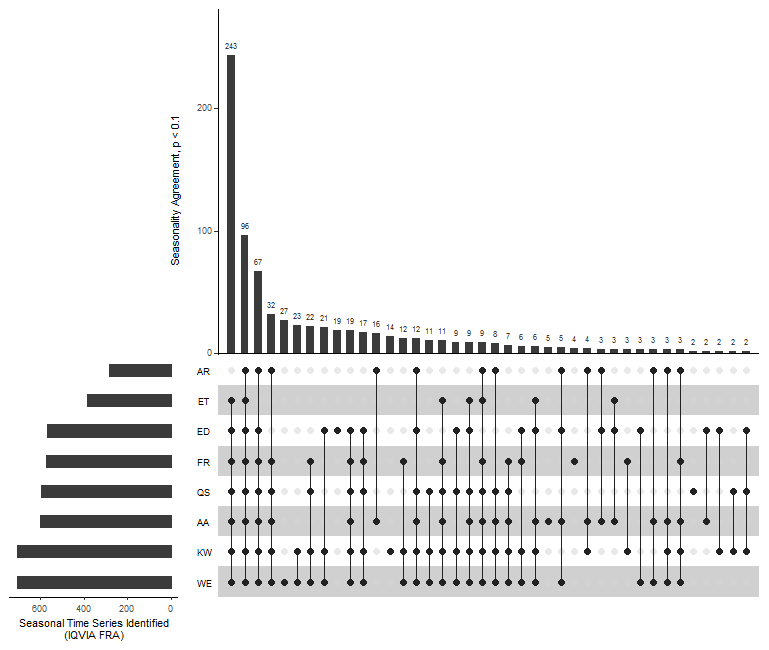

Supplement: Supplementary file 3 — Additional file 3: upsetRplots.zip. All 30 UpsetR plots. [file 12874_2022_1652_MOESM3_ESM.zip › upsetRplots/FRA1.png]

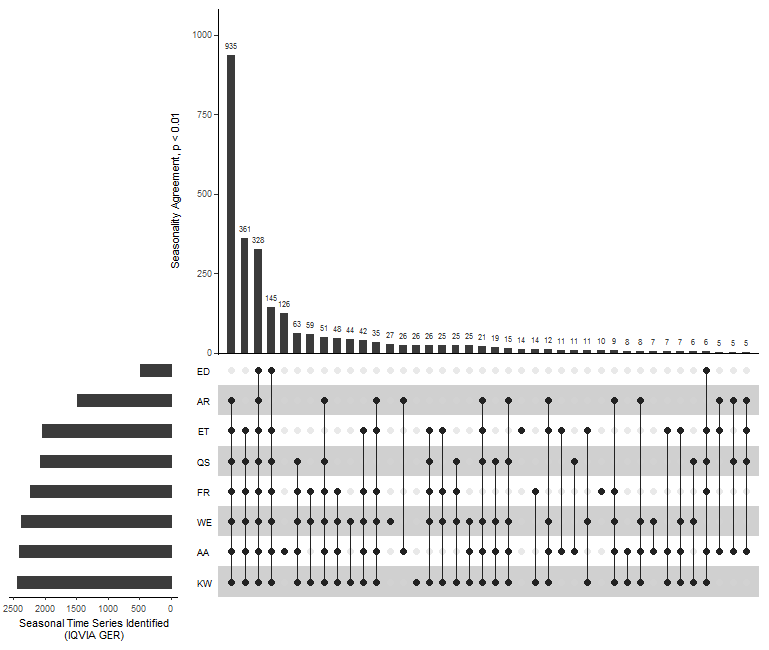

Supplement: Supplementary file 3 — Additional file 3: upsetRplots.zip. All 30 UpsetR plots. [file 12874_2022_1652_MOESM3_ESM.zip › upsetRplots/GER01.png]

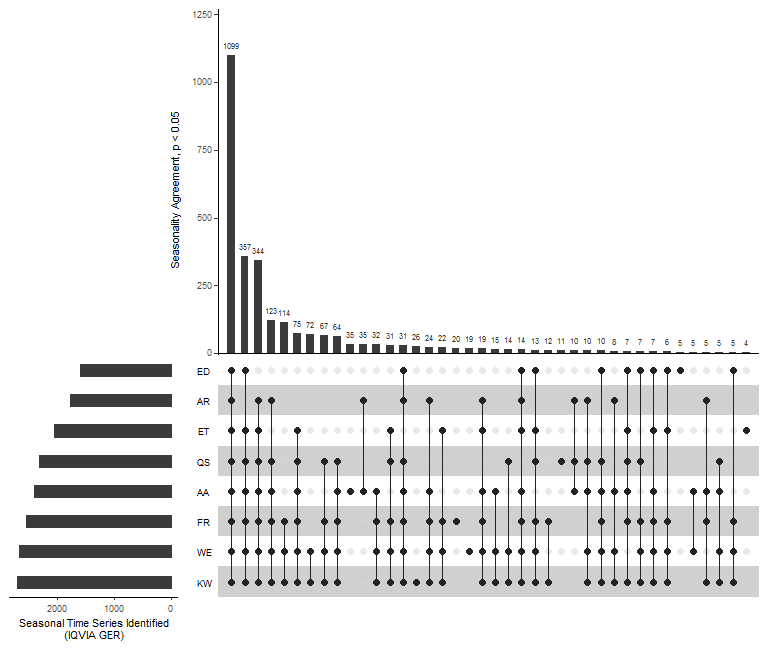

Supplement: Supplementary file 3 — Additional file 3: upsetRplots.zip. All 30 UpsetR plots. [file 12874_2022_1652_MOESM3_ESM.zip › upsetRplots/GER05.png]

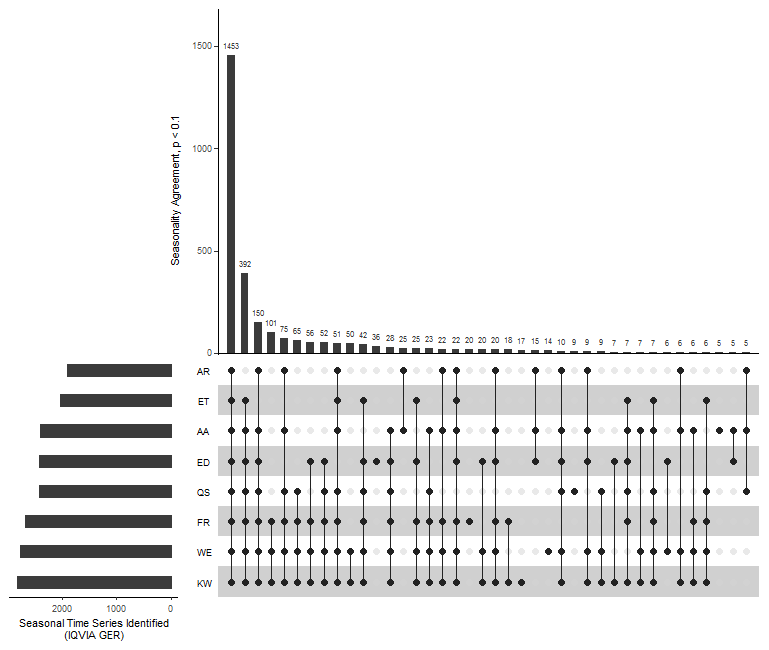

Supplement: Supplementary file 3 — Additional file 3: upsetRplots.zip. All 30 UpsetR plots. [file 12874_2022_1652_MOESM3_ESM.zip › upsetRplots/GER1.png]

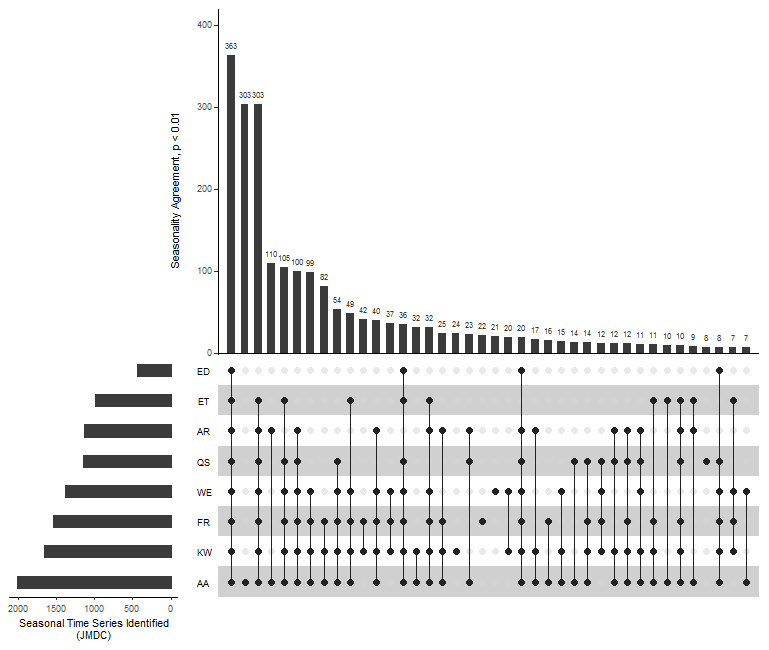

Supplement: Supplementary file 3 — Additional file 3: upsetRplots.zip. All 30 UpsetR plots. [file 12874_2022_1652_MOESM3_ESM.zip › upsetRplots/JMDC01.png]

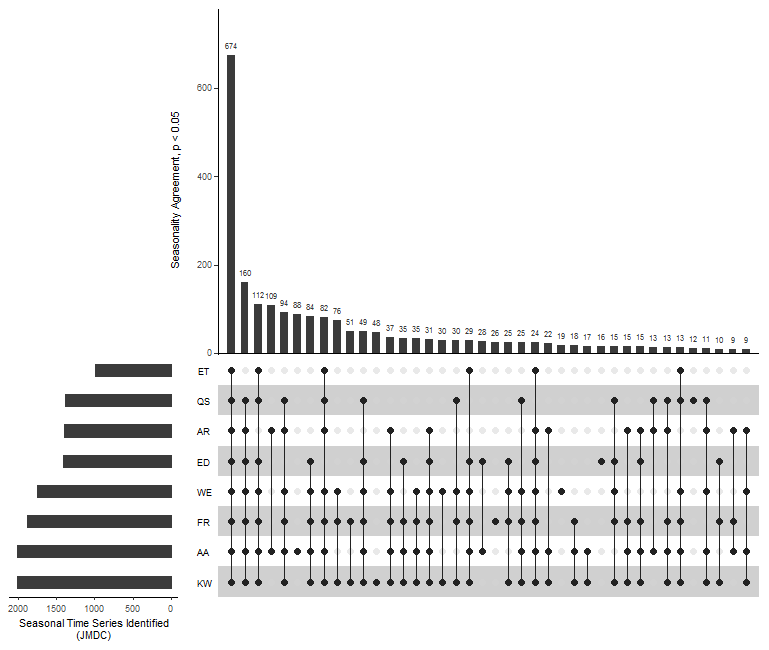

Supplement: Supplementary file 3 — Additional file 3: upsetRplots.zip. All 30 UpsetR plots. [file 12874_2022_1652_MOESM3_ESM.zip › upsetRplots/JMDC05.png]

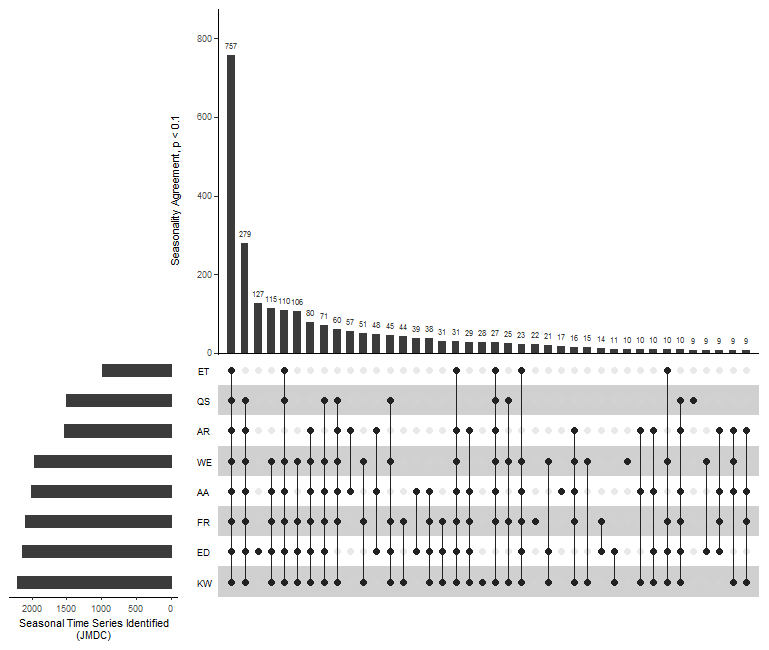

Supplement: Supplementary file 3 — Additional file 3: upsetRplots.zip. All 30 UpsetR plots. [file 12874_2022_1652_MOESM3_ESM.zip › upsetRplots/JMDC1.png]

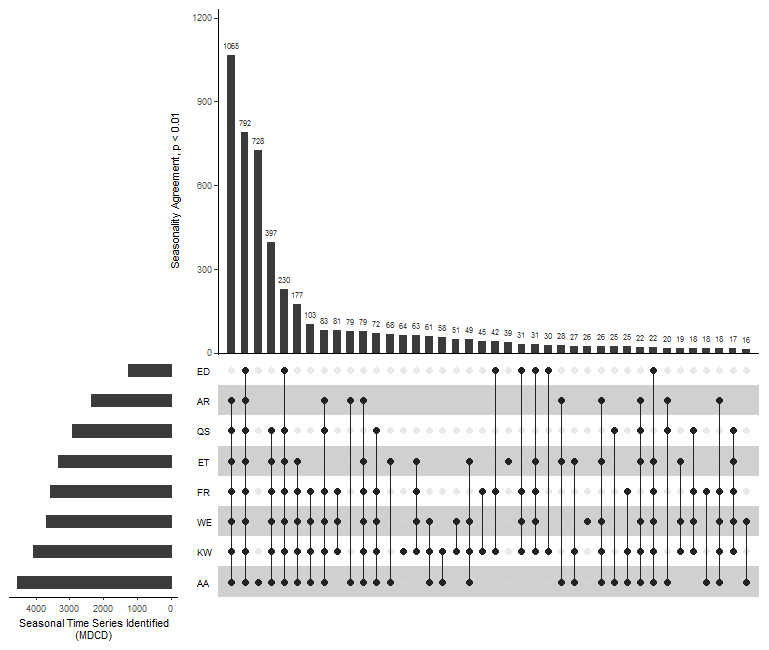

Supplement: Supplementary file 3 — Additional file 3: upsetRplots.zip. All 30 UpsetR plots. [file 12874_2022_1652_MOESM3_ESM.zip › upsetRplots/MDCD01.png]

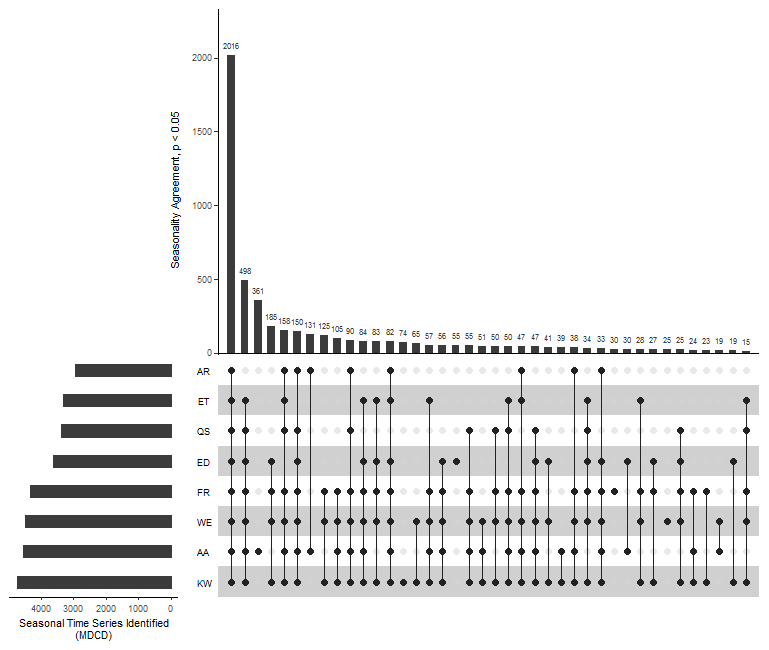

Supplement: Supplementary file 3 — Additional file 3: upsetRplots.zip. All 30 UpsetR plots. [file 12874_2022_1652_MOESM3_ESM.zip › upsetRplots/MDCD05.png]

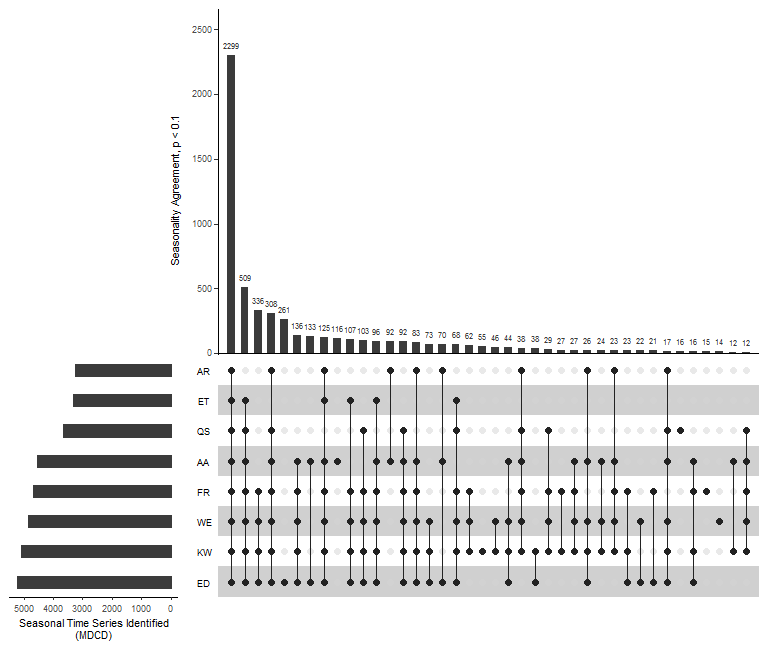

Supplement: Supplementary file 3 — Additional file 3: upsetRplots.zip. All 30 UpsetR plots. [file 12874_2022_1652_MOESM3_ESM.zip › upsetRplots/MDCD1.png]

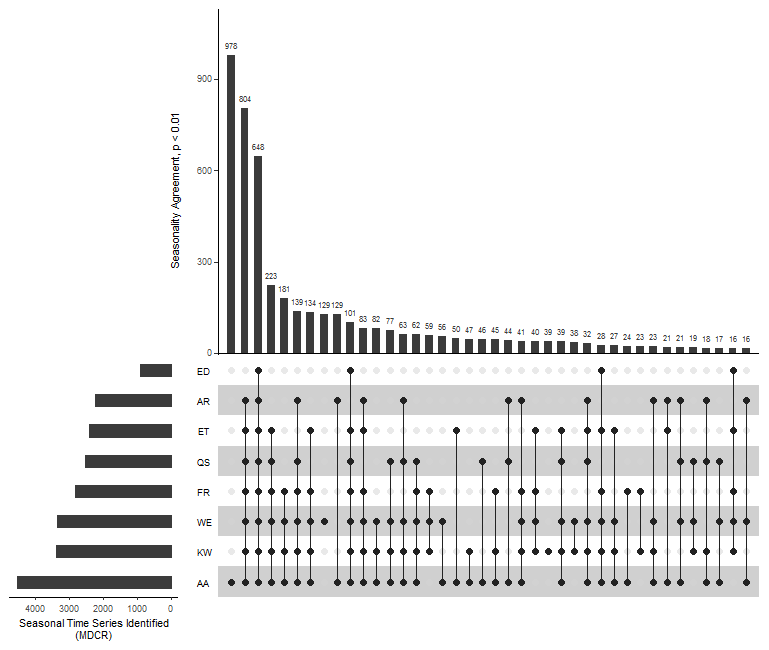

Supplement: Supplementary file 3 — Additional file 3: upsetRplots.zip. All 30 UpsetR plots. [file 12874_2022_1652_MOESM3_ESM.zip › upsetRplots/MDCR01.png]

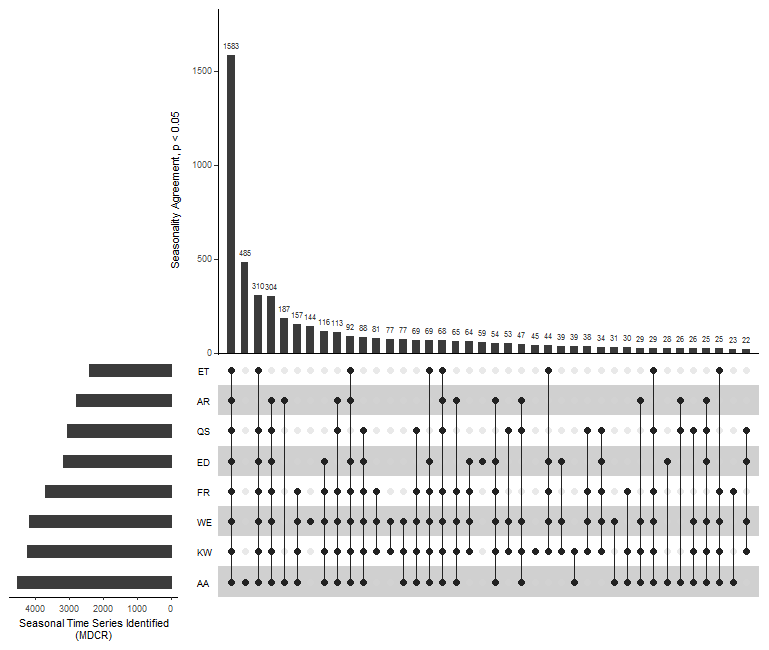

Supplement: Supplementary file 3 — Additional file 3: upsetRplots.zip. All 30 UpsetR plots. [file 12874_2022_1652_MOESM3_ESM.zip › upsetRplots/MDCR05.png]

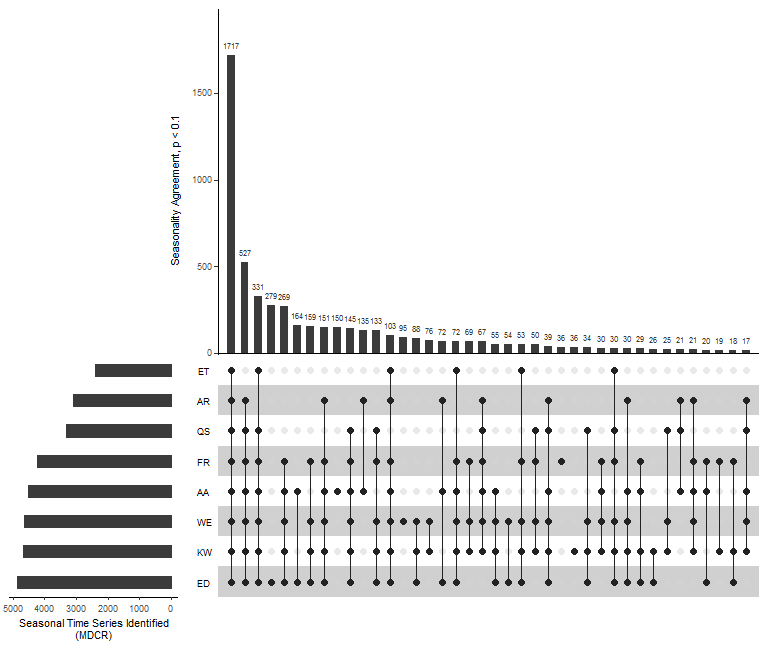

Supplement: Supplementary file 3 — Additional file 3: upsetRplots.zip. All 30 UpsetR plots. [file 12874_2022_1652_MOESM3_ESM.zip › upsetRplots/MDCR1.png]

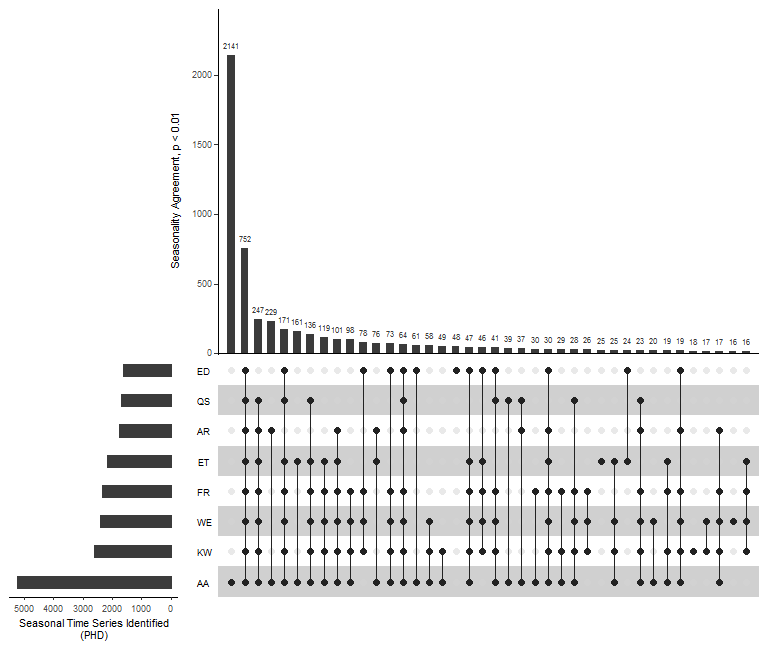

Supplement: Supplementary file 3 — Additional file 3: upsetRplots.zip. All 30 UpsetR plots. [file 12874_2022_1652_MOESM3_ESM.zip › upsetRplots/PHD01.png]

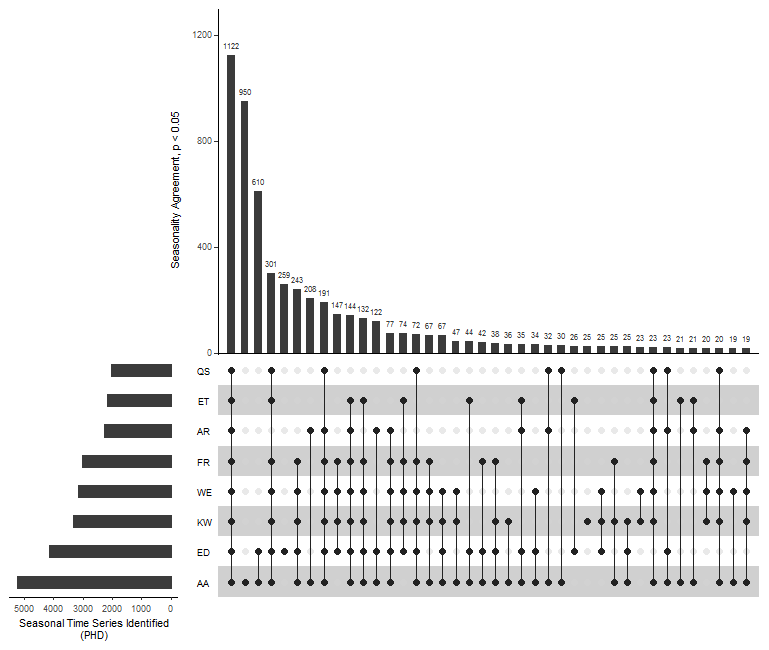

Supplement: Supplementary file 3 — Additional file 3: upsetRplots.zip. All 30 UpsetR plots. [file 12874_2022_1652_MOESM3_ESM.zip › upsetRplots/PHD05.png]

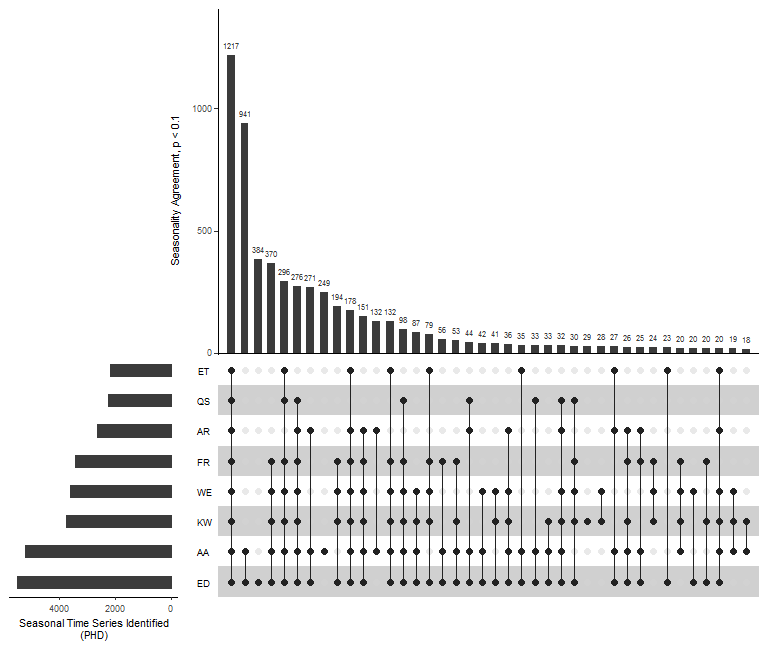

Supplement: Supplementary file 3 — Additional file 3: upsetRplots.zip. All 30 UpsetR plots. [file 12874_2022_1652_MOESM3_ESM.zip › upsetRplots/PHD1.png]
